# Comparison of Efficacy and Side Effects of Multispot Lasers and Conventional Lasers for Diabetic Retinopathy Treatment

**DOI:** 10.4274/tjo.75032

**Published:** 2017-01-17

**Authors:** Hande Çeliker, Azer Erdağı Bulut, Özlem Şahin

**Affiliations:** 1 Marmara University Faculty of Medicine, Department of Ophthalmology, İstanbul, Turkey

**Keywords:** Conventional lasers, diabetic retinopathy treatment, multispot lasers, side effects

## Abstract

Panretinal photocoagulation (PRP) is a standard treatment for proliferative diabetic retinopathy. Conventional laser (CL) therapy is performed in one or more sessions in single spot mode. Visual disabilities have been reported after treatment with CL, including central vision loss due to macular edema and peripheral visual field loss resulting from extensive inner retinal scarring. Multispot laser (MSL) photocoagulation has recently been introduced to clinical practice. Studies comparing PRP conducted with MSL and CL have reported that MSLs resulted in less retinal tissue damage and pain, and greater patient comfort compared to CL. The aim of this review was to compare the efficacy and side effects of MSLs and CLs for diabetic retinopathy treatment.

## INTRODUCTION

Panretinal laser photocoagulation (PRP) has been the gold standard for the management of proliferative diabetic retinopathy (PDR) since its efficacy was demonstrated in the Diabetic Retinopathy Study (DRS).^1^ PDR is performed with conventional laser (CL) over multiple sessions under local or topical anesthesia. The procedure is painful and time-consuming, which is tiring for both patients and physicians. As it requires multiple visits to an outpatient clinic, it also creates an additional load on retina clinics. Automated laser systems were developed in order to speed the photocoagulation process.^2,3^ However, the lack of constant physician control was also a disadvantage of these devices. A more recent innovation is the semiautomated multispot laser (MSL). These instruments allow multiple laser shots with a single pedal push, use frequency-doubled 532 nm Nd:YAG laser and are fully controlled by the physician.^4,5^ The aim of this review is to present an evaluation of the implementation, efficacy and side effects of the most recent generation of lasers currently in use.

## MULTISPOT LASERS

There are four MSLs in clinical use (Table 1).

## PATTERN SCANNING LASER

**Figure 1:**
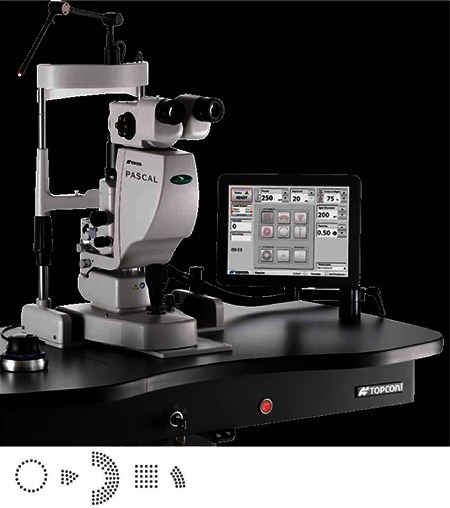
The PASCAL 532 nm instrument and panel showing available treatment patterns

The pattern scanning laser (PASCAL) is a semiautomated scanning laser application system that uses a frequency-doubled Nd:YAG laser to delivery multiple laser shots simultaneously to the retina, and was introduced to the market in 2006 (PASCAL® Laser, Optimedica Corp., Santa Clara, CA, USA) (Figure 1). The system can apply the laser as a single shot or as a 5x5 array, circle, arch, or line.^4^ As the pulse duration is much shorter (10-20 ms) compared to CL (100-200 ms) and multiple laser spots can be applied simultaneously, the procedure is faster and more comfortable for patients.^6,7^ The term semiautomated means that the physician has control at every stage of the procedure. Like older systems, the laser can be started and stopped using a foot pedal. As previously stated, other than its ability to deliver multiple or single laser shots, it is comparable to other CLs with similar features.^4^ There are many studies demonstrating the safety and efficacy of the PASCAL system.^8,9,10,11,12,13^

## VALON

**Figure 2:**
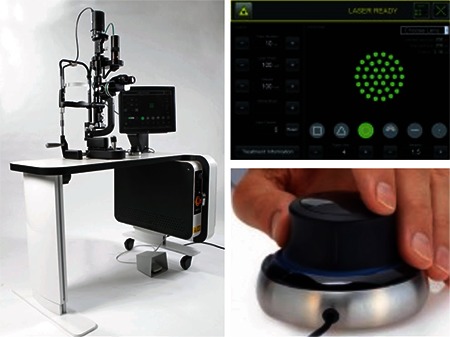
Valon laser instrument, screen and joystick

Like the PASCAL, the Valon MSL is also a semiautomated scanning laser system using a frequency-doubled Nd:YAG (532 nm) laser. The system is integrated into a Haag-Steit biomicroscope and is controlled via a touch screen and a joystick. Figure 2 shows a photograph of the device and its features are presented in Table 1. Various patterns, sizes, intensities and intervals can be selected on the screen and treatment settings can be adjusted as desired with the joystick.

As with the PASCAL, the spot number within a pattern can be adjusted from 1 to 36 depending on the pattern type and spot size. Valon’s most important feature, not shared by the PASCAL, is that the settings chosen with the joystick are displayed over the retinal image. This feature eliminates the need for physicians to look away from the microscope while making adjustments, thus saving time spent to focus back on the retina. Spot sizes of 50, 100, 200 or 300 μm can be selected from the microscope. Pulse duration can be adjusted to 10, 20 or 30 ms for multispot and up to 1,000 ms for single spots. The power can be increased up to 1,500 mW.^5^

## VISULAS 532S VITE

**Figure 3:**
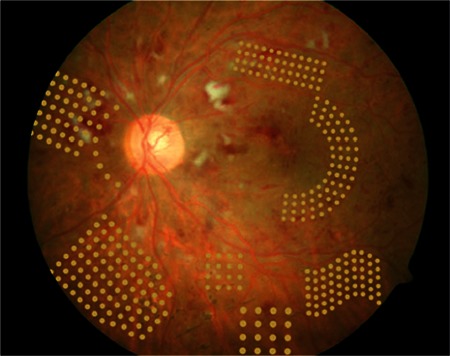
Multi-spot laser patterns available with the Visulas 532 VITE

The Visulas 532s VITE is a 532 nm solid-state laser system. Similar to the PASCAL and Valon systems, the Visulas 532s VITE can delivery laser as single spots or in preprogrammed multispot patterns. Linear or radial patterns are available. Selectable patterns are shown in Figure 3.

In a randomized, prospective study of 101 patients undergoing peripheral laser photocoagulation for various reasons, Röckl and Blum^14^ applied conventional single spot laser therapy in 35 patients (group A) and MSL therapy using the Visulas 532s VITE in 66 patients (group B). Spot size was consistent between the two groups (300 µm), while pulse duration was 100-150 ms for group A versus 20 ms for group B. Laser power was adjusted to produce moderate burns and the treatment time was recorded. After the procedure, patients were asked to rate their pain from 0 (painless) to 10 (maximum pain). Treatment time was shorter in group B than in group A. In group A, 46% of the patients reported pain at an average level of 4.4 (range, 2-8); in group B, only 1.3% of patients reported pain at a level of 3 or 4. The device’s features are summarized in Table 1.

## NAVILAS

**Figure 4:**
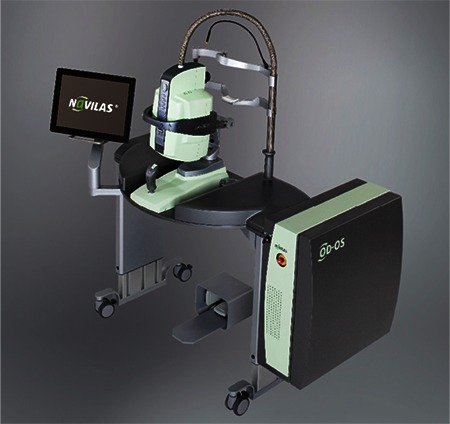
The Navilas system with integrated fundus camera

The Navilas laser photocoagulation system (OD-OS GmbH, Teltow, Germany), is a retinal navigation system and laser photocoagulation device including digital fundus imaging (live color fundus photography, red-free and infrared imaging and fluorescein angiography [FA]) (Figure 4). The instrument comprises an imaging camera, photocoagulation device (Merilas 532 nm) and a system that sends the laser beam from the ophthalmoscope to the target via moving mirrors. Its laser is a diode pumped solid-state laser (532 nm). The use of a fundus camera to aim the laser is a distinct feature from CLs and MSLs. This allows a larger glare-free field of view compared to a slit lamp. Because the displayed image is in the same format as that of an ordinary fundus camera, it is easier to implement a treatment plan based on the actual appearance. Treatment points, planned according to fundus photography or FA, are reflected on the live retinal image during treatment. This system was developed to allow the accurate localization of treatment to delicate lesions like microaneurysms and increase treatment efficacy. The device’s features are summarized in Table 1.

**Figure 5:**
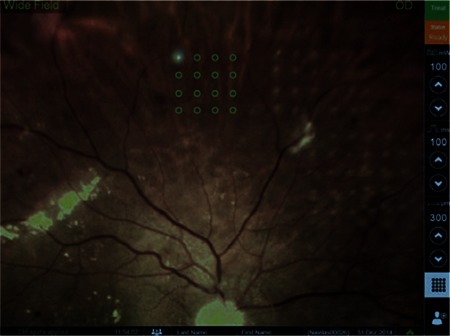
The Navilas screen

Another difference between this system and other slit-lamp laser devices is the touch screen used for visualization, planning and treatment (Figure 5). The retinal surgeon determines the laser application site using the screen and applies the laser in multispot or single spot mode. The surgeon manually actuates the laser after verifying the target lock.^15^

The laser spot qualities of the Navilas and PASCAL systems were compared in a study of PRP including 73 eyes of 51 high-risk PDR patients.^16^ Eyes underwent PRP with PASCAL or Navilas at pulse durations of 30 ms (16 and 21 eyes, respectively) or 100 ms (16 and 20 eyes, respectively). Laser spot size (major and minor diameters and area) and ellipticity (ratio of the major to minor diameter) were measured from fundus photographs taken from all quadrants 5 minutes after the procedure. Pain perception on a visual analog scale (from 0-10) was also compared. Burn size variation was 22% with 30-ms Navilas laser, 24% with 100-ms Navilas laser, 21% with 30-ms PASCAL pattern laser and 35% with PASCAL 100-ms single-spot laser. Nearing the equator, the Navilas showed less variation compared to the PASCAL (15% vs 25%). Toward the periphery, burn areas were more elliptical with the PASCAL, while Navilas spots were more uniform. Patients treated with 100-ms pulse durations reported less pain with the Navilas system than the PASCAL. Patients also reported less pain with the Navilas at 30 ms pulse duration, but the difference was not statistically significant. Previous studies using the Navilas have focused on the treatment of diabetic macular edema (DME). In a study published in 2011, 86 eyes of 61 patients with DR and DME were treated with Navilas; as a control group, 4 eyes of 4 patients were treated with standard manual laser.^17^ Pretreatment FA images marked with the treatment plan were overlaid on posttreatment color fundus photographs in order to measure efficacy. Analysis of 400 randomly selected focal spots showed that Navilas hit 92% of microaneurysm targets, while analysis of 100 focal spots from the control group showed an accuracy rate of 72%. In summary, the Navilas has been demonstrated reliable and more effective than standard techniques in laser photocoagulation.

## TREATMENT EFFICACY OF MULTISPOT LASERS

Guidelines regarding how and to what extent PRP therapy should be implemented and when it should be repeated were set forth in the Early Treatment Diabetic Retinopathy Study (ETDRS).^18^ Laser application is performed at pulse durations of 100-200 ms, spot size of 500 μm, and power ranging between 100 and 750 mW to produce gray-white burns. For PDR, a total of about 1,500 burns spaced one spot width apart are applied in an area from 1 optic disc (OD) width nasal to the OD and 2 disc widths temporal to the macula, extending to one spot width of the inferior and superior vascular arcades and the equator in the periphery.^18^ The procedure may be performed in one session under local (peribulbar) anesthesia, or in 2 or 3 sessions at 1-2 week intervals under topical anesthesia. Single-session (SS) therapy is reported to be less preferable due to a higher rate of side effects (associated with both PRP and local anesthesia).^19^

With the introduction of MSLs, SS therapy has become a viable option once more. Treatment time with MSLs is approximately one-fifth that required with CLs, resulting in less pain, less inflammation and thus a lower incidence of complications like macular edema.

Although no multicenter studies have been conducted to date, there are single-center studies from medical facilities using these systems. These studies have reported comparable efficacy and reliability between MSLs and CLs. Nagpal et al.^8^ performed PRP on 30 eyes using the PASCAL system and 30 eyes using a 532 nm CL. Both treatments were performed in two sessions. Patients underwent follow-up examination at 1, 3, and 6 months after treatment. Based on clinical findings and fundus imaging, both treatments were determined effective.

In a study by Muraly et al.^9^ comparing PASCAL and 532 nm CL, one eye of each patient was treated with SS-PRP using the PASCAL system (mean 2,795 spots), while the other eye was treated with multisession PRP (MS-PRP) using a CL over 2 or 3 sessions (mean 1,414 spots). SS-PRP was 90% effective and MS-PRP was 64% effective at 1 month; both were 98% effective at 6 months.

Muqit et al.^11^ studied 40 eyes of 24 patients with PDR. Half of the eyes were treated with 1,500 single spots at 100 ms duration using PASCAL over the course of 3 sessions at 2-week intervals; the other eyes were treated with 1,500 spots in a SS of 20-ms multispot laser. Twelve weeks after treatment, SS-PRP was 74% effective and MS-PRP was 53% effective, although the difference was not statistically significant.

Muqit et al.^20^ later retrospectively evaluated 36 eyes of 22 patients included in the abovementioned study. The patients, which had all undergone PRP with 1,500 100-ms or 20-ms PASCAL laser spots, were divided into 3 groups (mild, moderate, severe) based on their baseline PDR severity. Eyes that did not show PDR regression in later follow-up visits were treated with an additional SS of PASCAL PRP (top-up) therapy. They evaluated treatment efficacy after 18 months in patients for whom FA imaging was obtained using wide-field Optos® angiography. A total of 10 eyes (28%) exhibited complete PDR regression after one session of PRP. Top-up therapy resulted in PDR regression in 75% (n=6) of mild PDR cases, 67% (n=14) of moderate cases, and 43% (n=3) of severe cases. Mild PDR required an average of 2,187 burns, moderate cases required an average of 3,998 burns, and severe cases required an average of 6,924 burns to achieve complete PDR regression.

## EFFECT OF MULTISPOT LASER ON VISUAL FIELD

Diabetic patients may experience visual field defects due to severe nonPDR (NPDR) or PDR.^21^ The DRS and ETDRS both reported that visual field defects may worsen following laser therapy.^22,23^ In the ETDRS, visual field analysis was done at baseline and at 4 and 48 hours after treatment using Goldmann I-4e and I-2e test objects. I-4e was used to assess total score, I-2e was used to evaluate paracentral scotoma in the central 20 degrees. At 4 months, patients who had undergone full treatment had significantly more visual field loss compared to patients whose treatment was delayed (p<0.001). This loss was more moderate in cases with mild treatment. In a study comparing the effects of full PRP and mild PRP, both treatment methods caused comparable reductions in central visual field sensitivity. However, full therapy caused a markedly greater reduction in sensitivity in the peripheral visual field compared to mild therapy^24^. Muqit et al.^25^ evaluated the effect of argon laser PRP on the retinal nerve fiber layer (RNFL) and visual field in a study including 10 eyes. Visual field analysis of the central 10 degrees and 24 degrees using 24-2 SITA-fast at 10 weeks and 6 months post-treatment revealed improved mean deviation (MD) in a majority (8/10) of eyes.

In another study by Muqit et al.,^11^ 40 eyes of 24 patients with PDR were treated with 1,500 laser pulses, delivered to half of the eyes as 100-ms PASCAL spots in 3 sessions at 2-week intervals, and to the other half of the eyes as 20-ms PASCAL in a SS. Visual field analysis done 4 weeks after treatment showed significant improvement in MD compared to baseline in the 20-ms treatment group. They reported no significant change in the other group.

In a later study by Muqit et al.,^10^ areas with ischemia and retinal capillary nonperfusion on wide-field angiography were treated with 1,500 PASCAL laser burns with 20 ms pulse duration and 200 μm spot size through a Mainster 165 PRP lens. SITA-standard visual field analysis at 12 and 24 weeks post-laser showed a 1.25 dB improvement in MD.

Nagpal et al.^8^ compared CL and PASCAL in 60 patients who underwent PRP. They conducted visual field analysis at 1 month post-treatment and found that the eyes treated with PASCAL had higher retinal sensitivity, but the difference was not statistically significant.

None of the studies using Visulas 532s VITE®, Valon®, and Navilas® have evaluated the effect of PRP on visual field.

## EFFECT OF MULTISPOT LASERS ON RETINA NERVE FIBER LAYER THICKNESS AND CENTRAL MACULAR THICKNESS

Laser photocoagulation primarily affects the retinal pigment epithelium (RPE) and outer retinal layers. Examination of laser burns after 1 week reveals that laser therapy also causes edema in the inner retinal layers. Longer pulse durations have been reported to cause more pronounced edema compared to shorter durations.^13^ OCT studies have demonstrated that short pulse duration (20 ms) creates conical burns in the outer retinal layers, thus sparing the inner retinal layers.^26^ It has also been reported that high-power laser can cause full-thickness destruction of the retina, including the ganglion cell layer.^13^ Over time, ganglion cell damage can lead to reduced RNFLT and peripapillary RNFL thinning.

Blankenship^27^ reported thickening of the temporal RNFL following experimental laser photocoagulation in rabbits.

Muqit et al.^25^ applied 2,000 argon laser pulses at 100 ms duration, 300 μm spot size, and 136 mW power in multiple sessions (MS) to 10 eyes. They assessed RNFLT before and at 10 weeks and 6 months after laser therapy using time-domain (TD) OCT. They observed an 8 μm increase in RNFLT at 10 weeks (p<0.05) and a 4 μm decrease at 6 months (p<0.05) compared to baseline.

Eren et al.^28^ investigated the effect of PRP on CMT and RNFL by applying PRP to 52 eyes of 30 patients who were newly diagnosed with PDR and had undergone no previous treatment, then evaluating patients at 3 and 6 months post-laser. They noted marked RNFL thickening at 3 months, followed by a pronounced thinning compared to baseline at 6 months.

In a retrospective study of the effect of MSL on RNFLT, Park and Jee^29^ evaluated 33 eyes treated with PASCAL, 34 eyes treated with CL and 38 eyes that were not treated. Peripapillary RNFLT showed no significant changes at 6 months or 1 year in the PASCAL group but was markedly lower at both 6 months and 1 year in the CL group.

The effect of PRP on RNFLT was not evaluated in any of the Visulas 532s VITE®, Valon® or Navilas® studies.

In another study from our clinic which has not been published yet, mean RNFLT was 2.27 μm and 4.39 μm greater than baseline at 1 and 3 months, respectively, after 20-ms Valon laser therapy (p>0.05). Treatment with 100-ms CL resulted in a 3.74 μm increase in RNFLT at 1 month (p=0.03) and a 2.32 μm increase at 3 months (p=0.19).

Transient or persistent macular edema may occur after PRP. The DRS reported macular edema at 6 weeks post-treatment in 21% of eyes treated with argon laser and 46% of those treated with xenon arch.^30^

In a recent multicenter study by the Diabetic Retinopathy Clinical Research group, 155 eyes with NPDR or PDR were treated with 1,260-1,274 argon laser shots of 50-200 ms duration and 200-500 μm spot size. The physicians decided whether to apply the treatment in a SS or in 4 MS held at 4-week intervals. On day 3 after the SS or the first MS, CMT was increased by 9 μm in the SS group and 5 μm in the MS group. At 4 weeks, the increase was 13 μm in the SS group and 5 μm in the MS group, whereas the increase was equivalent (14 and 15 μm) in both groups at 17 weeks.^31^

In the previously discussed study by Muqit et al.^11^ including 40 eyes of 24 patients with PDR, CMT was also evaluated and the MS group showed 22 μm and 20 μm increases in CMT at 4 and 12 weeks, respectively (p<0.001). They reported no significant increase in CMT in the SS group.

In their previously mentioned study, Nagpal et al.^8^ noted no increase in macular thickness at 3 or 6 months in either study group. In a study by Muraly et al.^9^ comparing PASCAL and 532 nm CL, the authors reported that none of their patients developed macular edema.

In a study evaluating the effect of MSL on CMT, Watanachai et al.^32^ applied SS-PRP to 40 eyes newly diagnosed PDR with no prior treatment and central foveal thickness (CFT) <300 μm. They observed significant increases in CMT after 4 and 12 weeks (24 μm, p=0.001 and 17.4 μm, p=0.002, respectively). Two eyes developed macular edema at 12 weeks.

Oh et al.^33^ evaluated development rates and risk factors of macular edema after SS-PRP in 129 eyes with pre-treatment CFT <300 μm. Macular edema was noted in 11 eyes at 1 month after treatment; the edema had resolved in 5 of those eyes at 3 months. The formation of edema has been associated with the presence of subretinal fluid and retinal cystoid space on OCT.

CMT was not evaluated in any of the PRP studies using Visulas 532s VITE or Navilas.

## PAIN STUDIES WITH MULTISPOT LASERS

Laser photocoagulation is painful for some patients. This pain may result in some patients not completing their treatment. Various methods for pain prevention have been recommended in the literature (such as oral or topical nonsteroid anti-inflammatory drugs [NSAID] and peribulbar anesthesia).^34,35^ Possible causes of pain include thermal diffusion into the choroid, stimulation of the ciliary nerves in suprachoroidal space, thermal diffusion to the RNFL or direct thermal damage to the posterior ciliary nerves.

Al-Hussainy et al.^36^ conducted a prospective study in 20 patients indicated for PRP for various reasons. In a SS, they applied 500 CL shots with 0.1 s duration, 300 μm spot size to the superior or inferior region, and 500 CL shots with 0.02 ms duration, 300 μm spot size to the rest of the retina. Although greater power was required to induce moderate burns with 0.02 s pulse durations, pain assessment indicated that shorter durations caused less pain (1.41 for 0.02 s, 5.11 for 0.1 s).

Muqit et al.^7^ randomly applied 20-ms or 100-ms PASCAL PRP under topical oxybuprocaine to 40 treatment-naive eyes of 24 patients. A researcher blinded to the treatments used a pain questionnaire at 1 hour (numerical pain score [NPS]) and a headache questionnaire at 1 month (numerical headache score [NHS]). Mean NPS was 2.4 (mild) for the 20-ms group and 4.9 (moderate) for the 100-ms group; mean NHS was 1.5 for the 20-ms group and 3.2 for the 100-ms group. Both of the differences were significant.

In the previously mentioned study by Muraly et al.^9^ comparing PASCAL and a CL, patients were asked to rate their pain as mild, moderate, or severe after treatment. Patients reporting mild, moderate, and severe pain in the PASCAL group were 40, 10, and 11, while in the CL group these numbers were 11, 25, and 14, respectively.

Nagpal et al.^8^ performed PRP using PASCAL in one eye and a 532 nm CL in the fellow eye in 60 patients with bilateral symmetric PDR or severe NPDR. Following treatment, patients scored their pain using a visual analog scale (VAS). The average score was 4.6 in the CL group, compared to 0.33 in the PASCAL group.

Seymenoğlu et al.^37^ performed PRP in 70 PDR patients, half using PASCAL and half using a CL. Pain was scored 5 minutes after the procedure using the VAS. Mean pain score was 1.54±1.22 in the PASCAL group and 5.54±3.28 in the CL group, which was a statistically significant difference.

Pain assessment by VAS was also done in a study comparing the Navilas and PASCAL. Patients treated with 100-ms pulse durations reported less pain with the Navilas system than the PASCAL (1.0±0.91 vs 2.4±1.99). Patients also reported less pain with the Navilas at 30-ms pulse duration (0.9±1.14 vs 1.6±1.41), but the difference was not statistically significant.

In a randomized, prospective study by Röckl and Blum,^14^ 46% of the patients who underwent 100-ms single-spot peripheral laser photocoagulation with the Visulas 532s VITE reported pain at an average level of 4.4 (range, 2-8), while only 1.3% of those treated with 20-ms MSL reported pain at a level of 3 or 4.

Küçümen^38^ used the VAS to evaluate pain in 107 patients who underwent PASCAL photocoagulation for various reasons. Reported pain score distribution was 0 in 46%, 1 in 20.8%, 2 in 8.2%, 3 in 12.5%, and 4 in 12.5% of patients.

In a study from our clinic pending publication, 42 treatment-naive eyes of 21 patients underwent PRP. Each patient received 20 ms, 300 μm spot size Valon laser therapy in a SS or 100 ms, 300 μm spot size in 3 sessions. After each session, patients scored their pain using the VAS. Pain was in the mild range with both treatment methods (2.4 for 20 ms, 3.0 for 100 ms).

## CHOROIDAL DETACHMENT IN SINGLE-SESSION MULTISPOT LASER THERAPY

In their comparison of SS and MS argon laser PRP, Doft and Blankenship^19^ noted a significantly higher incidence of side effects such as exudative retinal detachment, choroidal detachment and elevated IOP in the SS group compared to the MS group. The PASCAL® laser has been used since 2006 and the Valon® laser and Visulas 532s VITE® since 2009 for SS-PRP. The aforementioned side effects have been reported in the literature as isolated cases. Of a series of 883 patients who underwent PASCAL SS-PRP within a period of 2 years, Natesh et al.^39^ observed symptomatic choroidal detachment in a single patient. Velez-Montoya et al.^40^ reported 2 cases of choroidal detachment and 1 case of exudative retinal detachment from among 1,301 patients who underwent PASCAL PRP in a span of 7 months. Sheth et al.^41^ noted choroidal detachment in 2 of 666 patients who underwent PASCAL PRP within a period of 2 years.

In another study from our clinic which will be published in the near future, we observed exudative retinal detachment in 1 eye from a series of 20 patients who underwent PRP using the Valon laser. The retinal detachment resolved within 15 days with topical NSAID and the patient experienced no reduction in visual acuity compared to baseline.

## CONCLUSION

PRP has been the gold standard in PDR treatment since it was proven effective in the DRS.^1^ PRP may be completed over MS conducted at intervals of 1-2 weeks. Completion in a SS is less common due to the need for more local anesthesia to manage the higher pain levels and the higher incidence of side effects such as macular edema, angle closure, and exudative retinal detachment. However, completion in MS requires a physician to spend more time per patient, requires the patient to make multiple trips to the hospital, and increases the economic burden of treatment. It also means that treatment remains incomplete in more patients due to poor patient compliance. SS CL therapy is not preferred by many physicians due to the higher incidence of side effects, the need for peribulbar anesthesia in most patients and possible complications related to anesthesia. However, dividing treatment into MS may delay the onset of treatment effect, particularly in high-risk PDR eyes. Complications of PDR may occur during the treatment period or before treatment takes effect. Because the complications that frequently arose due to CL MS-PRP are not a problem with new generation lasers, 20-ms SS-PRP with these lasers may be a favorable alternative to both SS and MS CL therapy. However, these patients must be followed and monitored in case further treatment is required.

The consensus among previous studies is that SS therapy with short duration MSL results in shorter treatment time and less pain compared to single spot therapy. However, as is evident from the above studies, it has not yet been determined whether the ETDRS-recommended number of burns is still applicable or whether more burns are required when applying 20-ms laser treatment with PASCAL® and similar systems like Valon®, Visulas VITE® and Navilas®. Patients who have undergone SS therapy with short duration MSL should also be monitored for treatment effect, and physicians should not hesitate to provide additional therapy when necessary.

### Ethics

Peer-review: Externally peer-reviewed.

## Figures and Tables

**Table 1:**
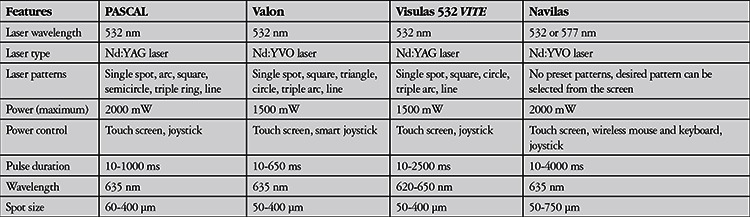
General features of multispot lasers
